# Factors influencing suicidal tendencies during COVID-19 pandemic in Korean multicultural adolescents: a cross-sectional study

**DOI:** 10.1186/s40359-022-00867-9

**Published:** 2022-06-21

**Authors:** Ju-Young Park, Insook Lee

**Affiliations:** 1 College of Nursing, Konyang University, Deajeon City, Republic of Korea; 2 Department of Nursing, Changwon National University, 20 Changwon Daehak-ro, Uichang-gu, Changwon, Gyeongnam 51140 Republic of Korea

**Keywords:** Adolescent, Mental health, Suicidal ideation, Suicide attempted, Risk factors

## Abstract

**Background:**

There is concern that the COVID-19 pandemic has had a negative impact on the psychological wellbeing of many populations, including increase of fear, anxiety, and uncertainty. Since the start of the COVID-19 pandemic, adolescents specifically have experienced direct and indirect impacts on their mentally, resulting in severe depression, self-harm and suicide. This study aimed to identify factors influencing suicidal tendencies and the mental health status of multicultural adolescents in Korea during the COVID-19 pandemic.

**Methods:**

This cross-sectional study was conducted with 784 multicultural adolescents (Korean fathers and foreign mothers) who participated in the 16th national Korean Youth Risk Behaviour online survey. Research variables were measured using self-reported questionnaires for mental health and suicidal tendencies. Data was analysed using SPSS 26.0 program.

**Results:**

The factors influencing suicidal tendencies (contemplating suicide, suicidal plans, and suicide attempts) were sexual intercourse experience (adjusted odds ratio [aOR], 7.67, 5.04, 7.10), depressive mood (aOR 1.03, 0.98, 0.97, 0.90), and unhappiness (aOR 13.00, 7.28, 5.56).

**Conclusions:**

In conclusion, the factors that affect suicidal tendencies showed sexual intercourse experience, depressive mood and unhappiness. Screening for suicidal tendencies and suicide prevention programs that consider the significant factors that affect suicidal tendencies should be developed for multicultural adolescents. School health professions and mental health counselors at schools need to emphasize the mental health and psychosocial support needs of senior high school students.

## Background

In the context of the current coronavirus disease-19 (COVID-19) pandemic, as of December 8, 2021, approximately 267,012,530 people worldwide were infected with COVID-19, and 5,269,852 had died [1]. People during the COVID-19 pandemic forced to be quarantined had higher odds of developing posttraumatic stress disorder (PTSD) symptoms than those voluntarily quarantined [2]. In the case of COVID-19, isolation, restricted movement, and stay-at-home measures to contain the spread of the infection have a particularly acute impact on women and children [3].

The chances of children being exposed to violence are dramatically increased as a result of pandemic-related restraints (spatial distancing, isolation, home quarantine, etc.), as family members spend more time in close contact and household stress intensifies, and the risk grows even greater when families also must cope with potential economic or job losses. These are leading to negative psychological reactions, such as fear, anxiety, loneliness, and uncertainty, and have a direct impact on mental and psychological health, including conflicts, family destruction, abuse, depression and domestic violence caused by the worsening domestic economy [4, 5]. Moreover, impacts on mental health (e.g., depression, anxiety, panic, and traumatic stress) can also occur due to the lack of accurate information regarding COVID-19 [6–8].

According to a study of 584 teenagers in China, 40.4% of the Chinese teenagers reported that they had psychological problems, and 14.4% said that they experienced posttraumatic stress syndrome symptoms two weeks after the outbreak of COVID-19 [9]. According to the Korean society for traumatic stress studies in 2020, during the COVID-19 pandemic in Korea, 14.2% of teenagers aged 13 to 18 years were at risk of severe depression, 11.2% were at risk of severe anxiety, and 10.2% had thought of self-injury or suicide within the past two weeks [10]. Previous studies have shown that a population’s mental and psychological reactions should not be overlooked during the management of an infectious disease pandemic [11]. Psychological reactions to pandemics include maladaptive behaviours, emotional distress, and defensive responses [12]. Psychological reactions to pandemics include maladaptive behaviors, emotional distress, and defensive responses, and these reactions are feared to cause disorders even after the end of a pandemic [12]. Furthermore, those who are prone to psychological problems are especially vulnerable to exhibiting reactions. It is important to pay attention to the mental health of teenagers who will have social and ecological impacts due to the psychological vulnerability of the entire family, school, and community environments.

The prevalence of infectious diseases such as COVID-19 can particularly affect mental health among adolescents who are socially and economically vulnerable or disadvantaged, while the type and degree of factors that affect their mental health may vary depending on various vulnerability factors [13]. For instance, poorer, younger, female students who were enrolled in lower year levels demonstrated higher levels of consequence related COVID-19 anxiety among students in the Philippines [14]. During pandemics, a particularly noteworthy victim is multicultural adolescents who fall into the categories of vulnerable groups. In Korea, the total number of elementary, middle, and high school students decreased, while the number of multicultural students tripled from 0.9% in 2013 to 2.8% in 2020 [15]. Multicultural teenager refers to juveniles who live and grow in two or more cultures, who is a children of a family of immigrants by international marriage in which one of the parents is a naturalized citizen of the Republic of Korea [16]. Multicultural teenagers have different experiences from non-multicultural teenagers in home and school environments, so they become stressed in the face of various difficulties and experience negative emotions such as anxiety and depression. It has been reported that this is likely to lead to suicidal thoughts [17, 18]. According to an analysis of data from the Korea Youth Risk Behavior Web-based Survey (KYRBS)/Youth Health Behavior in 2019, multicultural adolescents had significantly higher numbers of suicide plans and suicide attempts than non-multicultural adolescents, and they had 1.62 times more suicide attempts [19]. The death toll from suicide in Korea from 2000 to 2019 was 24.6 per 100,000 people (46% increase), the highest level among Organization for Economic Cooperation and Development (OECD) member countries [20]. In particular, the suicide attempt rate among teenagers has been steadily rising since 2015 and has firmly topped the list of causes of death among teenagers since 2011 [21]. If there is no effective treatment or support for suicide in adolescents, adolescents’ mental health problems can have a devastating impact on their lives. Because the mental health problems and negative experiences of multicultural adolescents can lead to various physical and mental disorders and affect quality of life throughout their lifetime [13], it is very important to identify the factors due to COVID-19 that affect the suicide tendencies of multicultural adolescents. Additionally, it is necessary to approach the mental health issues of multicultural adolescents from a different perspective than how mental health is addressed in non-multicultural adolescents.

The COVID-19 pandemic has raised concerns about the mental health of this generation of children, who are the first to be protected from the impacts of COVID-19 and at the same time, are partners in the COVID-19 response [22]. A comprehensive approach is needed to promote good mental health for all children, protect vulnerable children and take care of children facing the greatest challenges. While several studies have been conducted on direct and indirect effects on the mental health of multicultural adolescents thus far, the suicide trend of multicultural adolescents has not been identified in the COVID-19 pandemic situation. Therefore, this study examined the mental health conditions they experienced after the outbreak of COVID-19 and provides practical help to prevent suicide in multicultural adolescents by identifying what factors affect their suicidal tendencies. The results of this study will be important in preparing measures to improve the mental health of multicultural adolescents, a vulnerable population group in infectious disease pandemics.

## Methods

### Aims

This study aimed to examine the mental health status of multicultural adolescents during the COVID-19 pandemic and to identify the effects on suicidal tendencies. The specific aims were as follows: 1) to identify the mental health and suicidal tendencies of participants; 2) to confirm suicidal tendencies according to the general characteristics of the participants and their mental health status; and 3) to explore the impact factors on the participants’ suicidal tendencies.

### Study design and setting

This was a cross-sectional study conducted with nationwide online cross-sectional survey data. The KYRBS [23, 24] was established in 2005 by the Korea Centers for Disease Control and Prevention (KCDC). The KYRBS is an ongoing national cross-sectional survey that assesses health-risk behaviours among middle- and high-school students, monitors progress towards achieving national health objectives of Korea's National Health Plan [25] and provides data for the development and evaluation of school health policies and programmes in Korea. The KYRBS focuses on health-risk behaviours among adolescents.

### Participants and procedure

The subjects were middle- and high-school students who participated in the 16th (2020) KYRBS organized by the KCDC and the Ministry of Education [26]. This nationwide online survey is the Korean government approval statistics survey (Approval No. 117058) and has been carried out every year since 2005. The sampling method was used, the primary extraction unit was school, and the second extraction unit was class. For the first extraction, a sample school was selected by permanent random number extraction method by layer. In the second extraction, one class was randomly extracted from the selected sample school by grade. All students in the class selected as a sample class were surveyed, and students with long-term absences, exceptional children who were unable to participate in the survey on their own, and students with text literacy disabilities were excluded from the sample students. The population is a total of 2,631,888 students from 5,624 schools, with a sample of 54,948 students from 793 schools. It is a survey data that can represent Korean youth by prioritizing five middle and high schools for each of 17 cities and provinces after distributing the sample size to 400 middle schools and 400 high schools. In other words, a total of 793 schools (398 middle schools and 395 high schools) and 54,948 students participated in the survey, with 94.9% participating in the survey based on the number of students. The number of middle school respondents 28,961 and high school respondents 25,987 represented 1,326,523 for middle school students nationwide and 1,305,365 for high school students nationwide as of April 2020.

The steering committee, representing the Office of Education from 17 provinces of Korea, oversaw the survey (August 3 to November 13, 2020). The survey was comprised of questions concerning health-risk behaviours among adolescents, including tobacco use, alcohol use, obesity and weight control efforts, physical activity levels, dietary behaviours, injuries, sexual behaviours, mental health, oral health, personal hygiene, substance use, socioeconomic status, atopy and asthma, internet addiction, and violence. On the day of the survey, the survey support teacher led the sample class students to the Internet-enabled school computer room, assigning one computer per person and assigning them randomly. For the 2020 survey, some schools that had difficulties conducting surveys in computer rooms due to COVID-19 surveyed the students in classrooms under the supervision of their teachers with mobile devices (tablet PCs and smartphones). The entire course of the survey lasted 45 to 50 min in class. If the survey did not respond, there was no response to the item in the original data of the survey because it used an online survey system that did not move on to the following question, but no response to some questions occurred due to the cancellation of logical errors and abnormal values. The non-response rate of the item was lower than 2%, so the result was calculated without replacing questions with no responses.

In this study, 54,948 teenage students who were born in Korea and whose mothers' nationalities were foreign were included on the basis that 95% of the multicultural adolescents in Korea have foreign mothers and Korean fathers [27]. As a result, data from 784 multicultural adolescents (1.43%) were sampled and analysed.

### Measures

#### General characteristics

General characteristics of the subjects were measured using 10 questions on the following items: sex; visible minority status (if their mother’s country of birth was China, North Korea, Japan, Taiwan, or Mongolia, they was classified as an invisible minority); lifetime sexual intercourse; experience of being treated by a doctor due to violence (physical assault, intimidation, bullying, etc.); lifetime substance use; lifetime and monthly cigarettes and e-cigarette use; lifetime and current alcohol use; the perception of getting enough sleep; perceived health status; and perceived household economic difficulty after COVID-19.

#### Mental health

Mental health measures included depressive mood (feelings of sadness or hopelessness), loneliness, perceived stress, and perceived happiness. Depressive mood was measured using a binary scale: answering ‘yes’ to the question, ‘Have you ever felt sad or desperate enough to stop your daily life routines for two weeks in the last 12 months?’ was considered depression. Loneliness, which was measured with the question, ‘Have you ever felt frequently alone in the last 12 months?’, was reclassified into being alone (often alone, always alone) and not being alone (sometimes alone, not alone, not alone at all). Perceived stress, a degree that one normally feels, was reclassified into feeling stress (feeling much, feeling a lot, feeling a little) and not feeling stress (not feeling very much, not feeling at all). Perceived happiness, which is the subjective happiness status that one feels, was reclassified into happiness (very happy, happy), moderate happiness, and unhappiness (somewhat unhappy, very unhappy).

#### Suicidal tendencies

Suicidal tendencies measures consisted of items on contemplating suicide, suicidal plans, and suicide attempts. If one answered ‘yes’ to the question, ‘Have you ever seriously considered suicide in the last 12 months?’ was considered contemplating suicide. If one answered ‘yes’ to the question, ‘Have you ever made any specific plans to commit suicide in the last 12 months?’ was considered a suicidal plan. If one answered ‘yes’ to the question, ‘Have you ever attempted to commit suicide in the last 12 months?’ was considered a suicide attempt.

#### Ethical considerations

The KYRBS is a survey of nationally approved statistics (No. 117058) that is conducted annually by the Ministry of Education and the KCDC in Korea based on the National Health Promotion Act (Article 19). Students anonymously completed the self-administered questionnaire in a computer laboratory of each sampled school. The raw data are available to the public and were available for use after the data were officially approved for use through the KYRBS website. Additionally, the data were analysed after approval of deliberation exemption from the Institutional Review Board (IRB) of XX University.

## Statistical analysis

The data were integrated according to the raw data analysis guidelines, and the complex sample design elements were designated and analysed using the Statistical Package for Social Science (SPSS) for Windows program (version 26.0; SPSS Inc., Chicago, IL, USA). Descriptive statistical analysis (e.g., frequencies, percentages, means, and chi-square tests) was used for the general characteristics, mental health, and suicidal tendencies of the subjects. Pearson’s χ^2^ test was used to test the difference in suicidal tendencies according to general characteristics and mental health, and multiple logistic regression was performed to identify the factors affecting suicidal tendencies.

## Results

### General characteristics, mental health and suicidal tendencies of the subjects

The general characteristics, mental health and suicidal tendencies of the subjects are shown in Table 1. Of the total sample (N = 784; mean age = 14.38 ± 1.76 years), 47.4% were male students, 52.6% were female students, 51.8% were visible minorities, 48.2% were invisible minorities, 27.4% were cigarette and/or e-cigarette users, and 7.1% were alcohol users. Regarding perceived health status, 65.9% responded that they were healthy, and 39.3% responded that they had a difficult household economic status after COVID-19.

**Table 1:** General characteristics, mental health and suicidal tendencies **(N = 784)**

Variables	Categories	n (%) or Mean ± SD
*General characteristics*		
Age (years)		14.38 ± 1.76
Gender	Male	372 (47.4)
	Female	412 (52.6)
Visible minority	Visible	406 (51.8)
	Invisible	378 (48.2)
Sexual intercourse experience	Yes	30 (3.8)
	No	754 (96.2)
Experience of being treated by doctor due to violence	Yes	5 (0.6)
	No	779 (99.4)
Substance use	Yes	3 (0.4)
	No	781 (99.6)
Cigarette and/or e-cigarette use	Yes	215 (27.4)
	No	569 (72.6)
Alcohol use	Yes	56 (7.1)
	No	728 (92.9)
Perception of getting enough sleep	enough	274 (34.9)
	not enough	510 (65.1)
Perceived health status	Healthy	517 (65.9)
	Middle	197 (25.1)
	Poor	70 (8.9)
Perceived household economic difficulty after COVID-19	Yes	308 (39.3)
	No	476 (60.7)
*Mental health*		
Depressive mood	Yes	193 (24.6)
	No	591 (75.4)
Loneliness	Yes	392 (50.0)
	No	392 (50.0)
Perceived stress	Yes	599 (76.4)
	No	185 (23.6)
Perceived happiness	Happiness	478 (61.0)
	Moderate	232 (29.6)
	Unhappiness	74 (9.4)
*Suicidal tendencies*		
Contemplating suicide	Yes	72 (9.2)
	No	712 (90.8)
Suicidal plans	Yes	24 (3.1)
	No	760 (96.9)
Suicide attempts	Yes	18 (2.3)
	No	766 (97.7)

For mental health, 24.6% responded that they felt depressive mood, 50.5% responded that they felt loneliness, 76.4% responded that they felt a lot of stress, and 9.4% responded that they were unhappy. Seventy-two participants (9.2%) reported that they had contemplated suicidal during the COVID-19 outbreak, 3.1% reported having suicidal plans, and 2.3% reported suicide attempts.

### Suicidal tendencies according to mental health and general characteristics

Differences in suicidal tendencies according to mental health and general characteristics are shown in Table 2. Participants who contemplated suicide were more likely to (1) be female (χ^2^ = 10.63, *p* = 0.001), (2) be having sexual intercourse (χ^2^ = 28.25, *p* < 0.001), (3) be cigarette and/or e-cigarette users (χ^2^ = 5.44, *p* = 0.020), (4) be alcohol users (χ^2^ = 9.73, *p* = 0.002), (5) have insufficient sleep (χ^2^ = 11.66, *p* = 0.001), and (6) perceive having a poor health status (χ^2^ = 28.62, *p* < 0.001). Participants who contemplated suicide were more likely to also manifest a significant depressive mood (31.6% vs. 1.9%; χ^2^ = 154.34, *p* < 0.001), loneliness (16.6% vs. 1.8%; χ^2^ = 51.45, *p* < 0.001), perceived stress (11.7% vs. 1.1%; χ^2^ = 19.06, *p* < 0.001), and low perceived happiness (3.8% vs. 10.3% vs. 40.5%; χ^2^ = 104.44, *p* < 0.001).

**Table 2:** Differences in suicidal tendencies by general characteristics and mental health (**N = 784**)

Variables	Categories	Contemplating suicide		χ^2^ (*p*)	Suicidal plans		χ^2^ (*p*)	Suicide attempts		χ^2^ (*p*)
		No (n = 712)	Yes (n = 72)		No (n = 760)	Yes (n = 24)		No (n = 766)	Yes (n = 18)	
		n (%)	n (%)		n (%)	n (%)		n (%)	n (%)	
*General characteristics*										
Gender	Male (n = 372)	351 (94.4)	21 (5.6)	10.63^*^ (.001) ^†^	364 (97.8)	8 (2.2)	1.98 (.115) ^†^	366 (98.4)	6 (1.6)	1.47 (.244) ^†^
	Female (n = 412)	361 (87.6)	51 (12.4)		396 (96.1)	16 (3.9)		400 (97.1)	12 (2.9)	
Visible minority	Visible (n = 406)	371 (91.4)	35 (8.6)	0.32 (.621) ^†^	396 (97.5)	10 (2.51)	1.021 (.407) ^†^	399 (98.3)	7 (1.7)	1.23 (.342) ^†^
	Invisible (n = 378)	341 (90.2)	37 (9.8)		364 (96.3)	14 (3.7)		367 (97.1)	11 (2.9)	
Sexual intercourse experience	Yes (n = 30)	19 (63.3)	11 (36.7)	28.25^**^ (< .001) ^†^	26 (86.7)	4 (13.3)	11.09^*^ (.011) ^†^	26 (86.7)	4 (13.3)	16.94^*^ (.004)
	No (n = 754)	693 (91.9)	61(8.1)		734 (97.3)	20 (2.7)		740 (98.1)	14 (1.9)	
Experience of being treated by doctor due to violence	Yes (n = 5)	5 (100)	0 (0.0)	0.51 (.476) ^†^	5 (100)	0 (0.0)	0.16 (.690) ^†^	4 (80.0)	1 (20.0)	7.03 (.110) ^†^
	No (n = 779)	707 (90.8)	72 (9.2)		755 (96.9)	24 (3.1)		762 (97.8)	17 (2.2)	
Substance use	Yes (n = 3)	2 (66.7)	1 (33.3)	2.11 (.251) ^†^	2 (66.7)	1 (33.3)	9.30 (.089) ^†^	2 (66.7)	1 (33.3)	12.93 (.067) ^†^
	No (n = 781)	710 (90.9)	71 (9.1)		758 (97.1)	23 (2.9)		764 (97.8)	17 (2.2)	
Cigarette and/or e-cigarette use	Yes (n = 215)	46 (82.1)	10 (17.9)	5.44^*^ (.020)	55 (98.2)	1 (1.8)	0.33 (.565)	53 (94.6)	3 (5.4)	2.52 (.112)
	No (n = 569)	666 (91.5)	62 (8.5)		705 (96.8)	23 (3.2)		713 (97.9)	15 (2.1)	
Alcohol use	Yes (n = 56)	184 (85.6)	31 (14.4)	9.73^*^ (.002)	206 (95.8)	9 (4.2)	1.26 (.261)	205 (95.3)	10 (4.7)	7.33^*^ (.007)
	No (n = 728)	528 (92.8)	41 (7.2)		554 (97.4)	15 (2.6)		561 (98.6)	8 (1.4)	
Perception of getting enough sleep	Sufficient (n = 274)	262 (95.6)	12 (4.4)	11.66^*^ (.001)	271 (98.9)	3 (1.1)	5.49^*^ (.019)	270 (98.5)	4 (1.5)	1.31 (.252)
	Insufficient (n = 510)	450 (88.2)	60 (11.8)		489 (95.9)	21 (4.1)		496 (97.3)	14 (2.7)	
Perceived health status	Healthy (n = 517)	489 (94.6)	28 (5.4)	28.62^**^ (< .001)	507 (98.1)	10 (1.9)	6.57^*^ (.037)	511 (98.8)	6 (1.2)	9.35^*^ (.009)
	Middle (n = 197)	168 (85.3)	29 (14.7)		187 (94.9)	10 (5.1)		189 (95.9)	8 (4.1)	
	Poor (n = 70)	55 (78.6)	15 (21.4)		66 (94.3)	4 (5.7)		66 (94.3)	4 (5.7)	
Perceived household economic difficulty after COVID-19	Yes (n = 308)	275 (89.3)	33 (10.7)	1.43 (.233)	298 (96.8)	10 (3.2)	0.06 (.808)	299 (97.1)	9 (2.9)	0.89 (.346)
	No (n = 476)	437 (91.8)	39 (8.2)		462 (97.1)	14 (2.9)		467 (98.1)	9 (1.9)	
*Mental health*										
Depressive mood	Yes (n = 193)	132 (68.4)	61 (31.6)	154.34^**^ (< .001)	173 (89.6)	20 (10.4)	45.99^**^ (< .001)	178 (92.2)	15 (7.8)	34.23^**^ (< .001)
	No (n = 591)	580 (98.1)	11 (1.9)		587 (99.3)	4 (0.7)		588 (99.5)	3 (0.5)	
Loneliness	Yes (n = 392)	327 (83.4)	65 (16.6)	51.45^**^ (< .001)	369 (94.1)	23 (5.9)	20.80^**^ (< .001)	374 (95.4)	18 (4.6)	18.42^**^ (< .001)
	No (n = 392)	385 (98.2)	7 (1.8)		391 (99.7)	1 (0.3)		392 (100)	0 (0.0)	
Perceived stress	Yes (n = 599)	529 (88.3)	70 (11.7)	19.06^**^ (< .001)	576 (96.2)	23 (3.8)	5.18^*^ (.023)	582(97.2)	17 (2.8)	3.33 (.068)
	No (n = 185)	183 (98.9)	2 (1.1)		184 (99.5)	1 (0.5)		184 (99.5)	1 (0.5)	
Perceived happiness	Happy (n = 478)	460 (96.2)	18 (3.8)	104.44^**^ (< .001)	472 (98.7)	6 (1.3)	40.00^**^ (< .001)	474 (99.2)	4 (0.8)	36.67^**^ (< .001)
	Moderate (n = 232)	208 (89.7)	24 (10.3)		225 (97.0)	7 (3.0)		227 (97.8)	5 (2.2)	
	Unhappy (n = 74)	44 (59.5)	30 (40.5)		63 (85.1)	11 (14.9)		65 (87.8)	9 (12.2)	

^†^Fisher’s Exact test

^*^ *p* < .05, ^**^ *p* < .001

Participants with suicidal plans were more likely to (1) be having sexual intercourse (χ^2^ = 11.09, *p* = 0.011), (2) have insufficient sleep (χ^2^ = 5.49, *p* = 0.019), and (3) perceive having a poor health status (χ^2^ = 6.57, *p* = 0.037). Participants who had suicidal plans were more likely to also manifest significant depressive mood (10.4% vs. 0.7%; χ^2^ = 45.99, *p* < 0.001), loneliness (5.9% vs. 0.3%; χ^2^ = 20.80, *p* < 0.001), perceived stress (3.8% vs. 0.5%; χ^2^ = 5.18, *p* = 0.023), and perceived happiness (1.3% vs. 3.0% vs. 14.9%; χ^2^ = 40.00, *p* < 0.001).

Participants who attempted suicide were more likely to (1) be having sexual intercourse (χ^2^ = 16.94, *p* = 0.004), (2) be alcohol users (χ^2^ = 7.33, *p* = 0.007), and (3) perceive having a poor health status (χ^2^ = 9.35, *p* = 0.009). Participants who attempted suicide were more likely to also manifest significant depressive mood (7.8% vs. 0.5%; χ^2^ = 34.23, *p* < 0.001), loneliness (4.6% vs. 0%; χ^2^ = 18.42, *p* < 0.001), and perceived happiness (0.8% vs. 2.2% vs. 12.2%; χ^2^ = 36.67, *p* < 0.001).

### Factors affecting suicidal tendencies

The factors affecting suicidal tendencies are shown in Table 3. The general characteristics item for sexual intercourse, and the mental health items for depressive mood, loneliness and perceived happiness were statistically significant factors affecting the contemplating suicide (–2 Log likelihood = 514.97, Nagelkerke R^2^ = 0.47). The general characteristics item for sexual intercourse, and the mental health items for depressive mood and perceived happiness were statistically significant factors that affected suicidal plans (–2 Log likelihood = 313.31, Nagelkerke R^2^ = 0.29). The general characteristics item for sexual intercourse, and the mental health items for depressive mood and perceived happiness were statistically significant factors affecting suicide attempts (–2 Log likelihood = 285.59, Nagelkerke R^2^ = 0.39).

**Table 3:** Influencing factors of suicidal tendencies among Korean adolescents (**N = 784**)

Variables	Categories	Contemplating suicide			Suicidal plans			Suicidal attempts		
		OR	95% CI	*p*	OR	95% CI	*p*	OR	95% CI	*p*
*General characteristics*										
Gender	Female	1.17	0.60 ~ 2.29	.641	0.86	0.32 ~ 2.30	.760	0.81	0.25 ~ 2.67	.734
	Male^†^									
Visible minority	Visible	1.56	0.83 ~ 3.00	.168	0.83	0.32 ~ 2.20	.707	0.84	0.26 ~ 2.70	.767
	Invisible^†^									
Sexual intercourse experience	Yes	7.67	2.29 ~ 25.62	.001^*^	5.04	1.02 ~ 24.85	.047^*^	7.10	1.40 ~ 35.93	.018^*^
	No^†^									
Experience of being treated by doctor due to violence	Yes	0.00	0.00 ~ 0.00	.999	0.00	0.00 ~ 0.00	.999	22.98	0.64 ~ 830.24	.087
	No^†^									
Substance use	Yes	1.40	0.48 ~ 40.43	.845	17.02	0.22 ~ 13.67	.200	26.54	0.91 ~ 774.98	.057
	No^†^									
Cigarette and/or e-cigarette use	Yes	1.19	0.32 ~ 2.68	.887	0.17	0.02 ~ 1.60	.120	1.12	0.22 ~ 5.81	.890
	No^†^									
Alcohol use	Yes	1.18	0.59 ~ 2.42	.633	0.89	0.29 ~ 2.73	.838	1.59	0.43 ~ 5.90	.486
	No^†^									
Perception of getting enough sleep	Deprived	0.91	0.41 ~ 1.99	.806	1.41	0.37 ~ 5.38	.615	0.33	0.08 ~ 1.34	.121
	Sufficient^†^									
Perceived health status	Middle	1.95	0.97 ~ 3.93	.063	1.80	0.64 ~ 5.08	.265	2.20	0.60 ~ 8.04	.234
	Poor	2.13	0.87 ~ 5.20	.098	1.06	0.26 ~ 4.24	.940	3.22	0.64 ~ 16.22	.156
	Healthy^†^									
Perceived household economic difficulty after COVID-19	Yes	1.20	0.64 ~ 2.26	.569	1.34	0.52 ~ 3.43	.548	0.96	0.32 ~ 2.86	.936
	No^†^									
*Mental health*										
Depressive mood	Yes	13.00	6.22 ~ 27.18	< .001^**^	7.28	2.26 ~ 23.49	.001^**^	5.56	1.39 ~ 22.22	.015^*^
	No^†^									
Loneliness	Yes	2.66	1.07 ~ 6.65	.036^*^	8.08	1.00 ~ 68.33	.055	31.00	0.00 ~ 0.00	.993
	No^†^									
Perceived stress	Yes	1.67	0.35 ~ 7.93	.516	0.86	0.93 ~ 7.91	.892	0.33	0.31 ~ 3.42	.350
	No^†^									
Perceived happiness	Moderate	1.85	0.87 ~ 3.92	.018^*^	1.56	0.48 ~ 5.18	.472	2.36	0.52 ~ 10.65	.265
	Unhappy	5.58	2.42 ~ 12.87	< .001^**^	3.63	1.09 ~ 12.15	.036^*^	5.15	1.13 ~ 23.61	.035^*^
	Happy^†^									
Nagelkerke의 R^2^		.47			.34			.39		
Hosmer–Lemeshow (*p*)		3.74 (.880)			13.59 (.093)			2.17 (.975)		
Log likelihood (-2LL test)		289.44			149.03			108.73		

^†^Reference group; OR = adjusted Odds Ratio; 95% CI = 95% Confidence Interval

^*^ *p* < .05, ^**^ *p* < .001

In students with sexual intercourse experience, the rate of contemplating suicide was 7.67 times higher than that of students without sexual intercourse experience (OR, 7.67; 95% confidence interval [CI], 2.29∼25.62). Those who experienced depressive mood showed a 13.0-fold increase in contemplating suicide (OR, 13.00; 95% CI, 6.22∼27.18) compared to those who did not have depressive mood. Those who had loneliness showed a 2.66-fold increase in the contemplating suicide (OR, 2.66; 95% CI, 1.07∼6.65) compared to those who did not have loneliness. Regarding perceived happiness, those who replied as feeling ‘unhappiness’ showed a gradually higher risk of contemplating suicide (OR, 5.58; 95% CI, 2.42∼12.87) than those who replied as feeling ‘happiness’.

Students with sexual intercourse experience were 5.04 times more likely to have made suicidal plans than those without sexual intercourse experience (OR, 5.04; 95% CI, 1.02∼24.85). Those who had depressive mood showed a 7.28-fold increase in suicidal plans (OR, 7.28; 95% CI, 2.26∼23.49) compared to those who did not have depressive mood. Regarding perceived happiness, those who replied as feeling ‘unhappiness’ showed a gradually higher risk of suicidal plans (OR, 3.36; 95% CI, 1.09∼12.15) than those who replied as feeling ‘happiness’.

In the students with sexual intercourse experience, suicide attempts were 7.10 times higher than those without sexual intercourse experience (OR, 7.10; 95% CI, 1.40∼35.93). Those who had depressive mood showed a 5.56-fold increase in suicide attempts (OR, 5.56; 95% CI, 1.39∼22.22) compared to those who did not have depressive mood. Regarding perceived happiness, those who replied as feeling ‘unhappiness’ showed a gradually higher risk of suicidal plans (OR, 5.15; 95% CI, 1.13∼23.61) than those who replied as feeling ‘happiness’.

## Discussion

In the COVID-19 pandemic situation, national public health efforts are focused on combating pathogens rather than managing mental and psychological distress [28]. Suicide among adolescents is a serious problem worldwide, and the suicide rate of teenagers is increasing rapidly in South Korea. Therefore, as potential risk factors for the mental health of multicultural adolescents are likely to occur increase due to the COVID-19 pandemic, this study identified their mental health status and analysed factors that affect suicidal tendencies. The main research results are discussed as follows.

During the COVID-19 pandemic, multicultural adolescents were experiencing negative psychological emotions, with 51.9% who felt lonely and 77.7% who had perceived stress. The survey also showed that 24.6% and 9.4% felt depressive mood and unhappiness, respectively. As a result of analysing the same dataset of the 2020 KYRBS, 78.7% of teenagers were reported to perceive more than moderate stress [29], and 25.2% were reported to have experienced depressive mood [24], which is similar to the results of our study. According to Ko's findings [30], the mental health level of adolescents does not differ depending on whether they are multicultural adolescents, which is consistent with our study. However, while 14.1% of non-multicultural teenagers are reported to feel lonely [29], our study showed that more than half of multicultural teenagers feel lonely, so it is important to understand the cause of their loneliness before developing suicide prevention programs.

A longitudinal study of Americans [31] found that teenagers who considered committing suicide at the age of 15 were 12 times more likely to commit suicide when they turned 30. This shows that the idea of suicide in adolescence can lead to suicide in adulthood. Therefore, the painful long-term distress they experience should be recognized as a serious social problem. In particular, governments/policymakers should focus on preventing suicide by understanding the characteristics of multicultural adolescents and exploring internal and external environmental impact factors at home and school. The percentage of multicultural adolescents in Korea who contemplated suicide was 15.8% in the 2015 KYRBS data analysis [32], while the results of our study in 2020 showed a decreased percentage.

However, compared to all the teenagers who participated in the 2020 KYRBS [24], 10.9% of the adolescents contemplated suicide, 3.6% had made suicidal plans, and 2.0% had attempted suicide, indicating that the percentage of contemplating suicide (9.2%) and suicidal plans (3.1%) among multicultural adolescents is lower than that of all teenagers, but the suicide attempts (2.3%) percentage shows that the number of multicultural adolescents attempting suicide is higher than that of all teenagers. In other words, it can be confirmed that the risk index for attempted suicide by multicultural adolescents during COVID-19 was higher than that of ordinary teenagers. When controlling for other influencing factors, social support was a major factor influencing teenagers’ suicidal thoughts [33]. The social support of primary caregivers, intimacy with neighbours, the number of social support resources, and the unrelated racial social support of teenagers are correlated with a decline in suicidal thoughts [34]. Therefore, early intervention for multicultural adolescents should be made to prevent suicide attempts by establishing a social support system for families, schools and communities.

The factor that commonly affected the suicidal tendencies of multicultural adolescents was sexual intercourse experience. The odds ratios of contemplating suicide, suicidal plans and suicide attempts of multicultural teenagers with sexual intercourse experience were 7.67 times, 5.04 times and 7.10 times those of multicultural adolescents without sexual intercourse experience, respectively. According to an analysis of the KYRBS data from 2014 to 2016 [35], multicultural teenagers' suicidal thoughts, suicidal plans, and suicide attempts were higher than those of non-multicultural teenagers, and adolescents from multicultural families did not have higher rates of suicidal thoughts and suicidal plans than non-multicultural teenagers without sexual intercourse experience. Considering that sexual intercourse experience is higher in multicultural teenagers [19, 36], school nurses/teachers and mental health experts should recognize sexual experience as an important mediator and should make efforts to analyse negative mental health and prevent suicide by multicultural adolescents with sexual experience. It is also necessary to provide practical education to express sexual self-determination in adolescence when the possibility of sexual experience increases and to establish a multidimensional environment to seek advice and help with undesirable sexual experiences [37].

Suicidal attempts of teenagers who had been treated by a doctor due to violence were not significantly different from those who had not been treated for violence. According to an analysis of the 2014 KYRBS data [17], being treated by doctor due to violence was found to be a significant factor of suicidal attempts for Korean teenagers, but it was not noted in multicultural teenagers. However, the 2018 KYRBS data analysis showed that suicide attempts were 7.38 times higher in teenagers who had been treated by a doctor due to violence [18]. According to the 2015 KYRBS data analysis, teenagers were 2.39 times more likely to commit suicide in cases of repeated violence [38], and the more multicultural teenagers suffered from school violence, the more they thought of suicide [39]. Additionally, according to the 2015 KYRBS data analysis, multicultural teenagers' experience of violence was a very significant factor in suicidal thoughts [32] and had a significant influence on both suicidal plans and suicide attempts in line with suicidal thoughts [33]. Our results are different from previous studies because only 0.6% of the multicultural teenagers had been treated in a hospital due to violence, and we think our results should be supplemented to respond more specifically to adults who are the subject of violence with the question, "Have you received hospital treatment for violence (physical assault, intimidation, bullying, etc.) by friends, seniors and adults in the past 12 months?" to specifically understand the experience of violence by multicultural teenagers.

There was no significant difference in suicidal tendencies between the adolescents who experienced substance use and adolescents who did not. However, adolescents who experienced substance use in other studies analysing 2020 KYRBS data [40] had a 2.31 times higher risk of suicide contemplation than those with no experience, and in Jang’s study [19], the experience of substance use of multicultural adolescents was 3.39 times higher than that of non-multicultural adolescents [19]. Our study showed that multicultural adolescents who were habitual substance users had a low rate at 0.4%, but it is necessary to continue social attention even if there are a few multicultural adolescents with problematic behaviour.

Sex, cigarette and/or e-cigarette use, alcohol use, the perception of getting enough sleep, perceived health status, and perceived household economic difficulty after COVID-19 were not significant factors for suicidal tendencies. However, there were significant differences in sex, cigarette and/or e-cigarette use, and alcohol use in contemplating suicide, the perception of getting enough sleep in contemplating suicide and suicidal plans, and perceived health status in contemplating suicide, suicidal plans, and suicide attempts. This is difficult to interpret as a direct relationship between general characteristics and suicidal tendencies, but through prior studies, it can be inferred that it is related to suicidal tendencies between these variables. A previous study showed a high risk of suicidal thoughts among female students, teenagers who used cigarette and/or e-cigarettes and alcohol, and those with a low perception of getting enough sleep [40], low perceived health status and alcohol use showed a high risk of suicidal thoughts among multicultural adolescents [32]. After all, the individual and family environments were found to be related to the risk of suicide. Therefore, to reduce the suicidal tendencies of teenagers, an integrated program should be developed and applied to expand health care support projects linked to home-school communities, especially to improve female students' health behaviours, including cigarette and/or e-cigarette use, alcohol use, and sleep problems, and to improve their perceived health status, rather than limiting community programs only in consideration of multicultural characteristics.

The differences in suicidal tendencies according to mental health were significant in depressive mood, loneliness, perceived stress and perceived happiness. In other words, the rate of suicidal tendencies was found to be high when they felt depressed and lonely, perceived stress levels were high, and perceived happiness was low. This suggests that the mental health of multicultural adolescents is a problem that poses a risk of suicidal tendencies in itself, regardless of what other factors mediate it. Therefore, it is necessary to develop a school-based health education and counselling system that is focused on mental health, a step that should be performed first to detect high-risk groups of adolescents with suicidal tendencies at an early stage.

The mental health factor that commonly affected suicidal tendencies (contemplating suicide, suicidal plans, and suicide attempts) was depressive mood. According to an analysis of 2015 KYRBS data, suicidal thoughts of multicultural teenagers increased by 18.87 times as depressive mood increased [32], and depressive mood was a significant factor in suicidal thoughts, suicidal plans, and suicide attempts [35]. As contemplating suicide, suicidal plans, and suicide attempts are a sequential series, it is necessary to develop specific strategies to identify the causes and situations of depressive mood experienced by multicultural teenagers and focus on the psychology of their sadness and despair to block the connection. Family solidarity buffers the impact of adolescent depression on suicide risk [41], and living with family members affects suicidal tendencies [42]. It is therefore necessary to recognize the importance of the family, which is the most primary approach, and draw support from families and parents. According to the 2017 Multicultural Adolescents Panel Study [27], with participants whose fathers were Korean and whose mothers were foreign, the factors that commonly affected the depression of multicultural teenagers in both males and females were self-esteem, social interaction and school life satisfaction. In particular, for boys, depression was higher in those with low family support, poor relationships with their friends, mothers with cultural adaptation stress, and poor perceived health status; for girls, depression was higher in those with parents’ who were indifferent and had negligent parenting. Therefore, depression prevention programs to alleviate suicidal tendencies should be centred on self-esteem, social interaction and school life satisfaction, which are found to be common impact factors for both boys and girls, and can predict the effectiveness of intervention programs if the contents of the interventions vary depending on sex.

Our research, like previous studies [40, 43], showed that contemplating suicide is significantly higher when teenagers often or always feel lonely and are unhappy. In a study of teenagers aged 13 to 15 years and aged 16 to 17 years from 90 countries who attended the Global School-based Student Health Service (GSHS), common bullying or a lack of friends were reported to be associated with suicide accidents and suicide attempts [44], so interventions that focus on improving social relationships among teenagers are needed. In addition, loneliness is a variable in predicting suicidal thoughts, and hope is a variable that mediates them [45]; therefore, suicide prevention programs should be developed and applied to help reduce loneliness and increase hope.

Perceived stress was found to be a significant difference in suicidal tendencies but not an impact factor. A previous study showed that the perceived stress of multicultural teenagers explained 25% of suicidal thoughts with depression [39], while among multicultural teenagers, male students who were stressed from problems with their friends were 2.70 times more likely to commit suicide, and female students under stress from conflicts with their parents were 2.50 times more likely to commit suicide [46]. We believe there will be differences in research results because in the previous study, the subjects were defined as multicultural adolescents whose parents were not born in Korea, while our study defined the mother as a foreigner. In addition, since few teenagers with suicidal tendencies in our study said they had little stress, perceived stress may not have affected their suicidal tendencies. Nevertheless, according to a longitudinal study of teenagers in Canada [34], in the case of constant stress in childhood, the reported rate of suicidal thoughts in the around the age of 15 was 2.66 times higher than that of those who did not. Considering these results, we cannot rule out the long-term effects of sustained perceived stress on suicidal tendencies. Stress is also a factor that directly affects suicidal thoughts, but it is also a factor that mediates suicidal thoughts through mental health, such as depression and anxiety [47]. Our study and previous studies suggest that further research is needed to identify the mediating effects to explore various variables that affect suicidal tendencies.

Mental health is recognized as an important priority on the agenda of the United Nations (UN)'s Sustainable Development Goals (SDGs). The WHO emphasized the importance of mental health by proposing the Mental Health Action Plan (2013∼2030), which reduces each country's suicide rate by one-third by 2030 in line with the SDGs [3]. Knowing the mental health status of adolescents who will become world leaders in the future and the effects of suicidal tendencies is an important step towards this goal. Our study is meaningful in that it examined the mental health of multicultural adolescents and verified factors that affect suicidal tendencies in the COVID-19 pandemic situation. Our findings are expected to serve as an empirical basis for the development of suicide prevention programs for multicultural adolescents as the COVID-19 outbreak is prolonged. Additionally, the prolonged COVID-19 pandemic may have a significant impact on pre-COVID-19 mental health conditions and may deteriorate rapidly, further studies are recommended for adolescents with pre-COVID-19 psychiatric history.

## Limitations

The study used national data collected periodically by the government, so it can be said that the representative of respondents to Korea's youth mental health has been secured. However, the limitation of this study is that the validity and feasibility of the survey scale have not been clearly assessed. Therefore, the effectiveness of the tools needs to be verified, and it is necessary to apply the tools that are valid in the future. However, despite these limitations, the data analysed in this study can be generalized because random stratifying samples were used for teenagers across the country in Korea.

## Conclusions

As a result of this study, the sexual intercourse experience and depressive mood of multicultural adolescents are not related to problem behaviour, but they were found to be factors that can increase suicidal tendencies. These results showed that there are concerns about potential future increases in suicidal tendencies of multicultural adolescents and exacerbated mental health across the COVID-19 pandemic. The findings here also suggest the need for ongoing surveillance of how these risk factors for suicidal tendencies may be exacerbated by COVID-19 pandemic and as public health measures such as social distancing continue.

The results suggest the importance of implementing practical strategies to try to reduce contemplating suicide from leading to suicidal plans and suicide attempts through early detection and professional treatment of the health and psychological problems of multicultural adolescents. Sexual intercourse experience, depressive mood and perceived happiness are important and strong predictors of future suicide risk, so identifying modifiable risk factors for suicide tendencies that can be addressed through public health interventions during the pandemic and beyond is vital. Therefore, the school healthcare providers should make active efforts, such as supporting high-risk group screening programs and strengthening psychological and emotional interventions, to prevent suicide at the family, school, and community levels.

## Data Availability

The datasets used and/or analyzed during the current study are available from the corresponding author on reasonable request.

## Abbreviations

CI: Confidence interval
COVID-19: Coronavirus disease-19
GSHS: Global school-based student health service
IRB: Institutional review board
KCDC: Korea centers for disease control and prevention
KYRBS: Korea youth risk behavior web-based survey
OECD: Organization for economic cooperation and development
PC: Personal computer
PTSD: Posttraumatic stress disorder
SDGs: Sustainable development goals
UN: United nations
WHO: World health organization
